# Cardiac adverse events associated with quetiapine: Disproportionality analysis of FDA adverse event reporting system

**DOI:** 10.1111/cns.14215

**Published:** 2023-04-10

**Authors:** Yamin Shu, Yiling Ding, Lulu Liu, Qilin Zhang

**Affiliations:** ^1^ Department of Pharmacy Tongji Hospital, Tongji Medical College Huazhong University of Science and Technology Wuhan China; ^2^ Graduate School of Pharmaceutical Sciences University of Tokyo Tokyo Japan; ^3^ School of Pharmaceutical Science and Technology Tianjin University Tianjin China; ^4^ Department of Pharmacy Union Hospital, Tongji Medical College Huazhong University of Science and Technology Wuhan China

**Keywords:** cardiac adverse events, data mining, disproportionality analysis, FAERS, pharmacovigilance, quetiapine

## Abstract

**Objective:**

Quetiapine, an atypical second‐generation antipsychotic drug, is approved for treatment of schizophrenia, bipolar disorder, and depression. Due to the limitations of clinical trials, the association between quetiapine and rare cardiac adverse events (AEs) is still unclear. This study is to evaluate quetiapine‐associated cardiac AEs through data mining of FDA Adverse Event Reporting System (FAERS).

**Methods:**

Reporting odds ratio (ROR) was used to quantify the signals of quetiapine‐related cardiac AEs from the first quarter (Q1) of 2018–2022 Q1. Serious and nonserious cases were compared, and signals were prioritized using a rating scale.

**Results:**

A total of 1004 cases of quetiapine‐associated cardiac AEs were identified, with 31 signals being detected, among which 13 AEs were identified as new and unexpected signals. Besides, nine and 22 cardiac AEs were identified as moderate and weak clinical priority. The median TTO for moderate and weak clinical priority signals were 0 and 4 days, respectively. All of the cardiac AEs had early failure type characteristics, suggesting that most of the patients developed cardiac AEs in a few days after quetiapine treatment, and that the risk of cardiac AEs occurrence would be gradually decreased over time.

**Conclusion:**

Our study provided valuable evidence for health‐care professionals to mitigate the risk of quetiapine‐associated cardiac AEs based on an extensive analysis of a real‐world, large‐sample FAERS database, and prioritize cardiac AE signals.

## Graphical abstract

A total of 1004 cases of quetiapine‐associated cardiac adverse events (AEs) were detected, with 13 AEs were identified as new and unexpected signals. Besides, nine and 22 cardiac AEs were identified as moderate and weak clinical priority.

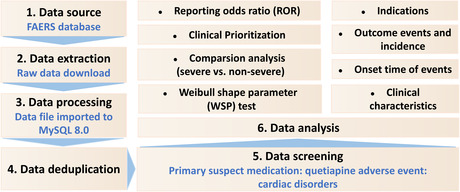

## 1 INTRODUCTION

Quetiapine, an atypical second‐generation antipsychotic drug, is approved by US Food and Drug Administration (FDA) for the treatment of schizophrenia, bipolar disorder, and depression. The antipsychotic activity of quetiapine is achieved by blocking central dopamine D_2_ receptors and serotonin 5‐HT_2_ receptors.^1^ According to the 2018 annual report of the American Association of Poison Control Centers based on the National Poison Data System, antipsychotics are the second largest group of toxic drugs in adults after painkillers, and quetiapine is one of the most frequently prescribed second‐generation antipsychotics.^2^ In recent years, an increasing number of new or serious adverse events (AEs) have been reported related to the long‐term use of quetiapine. Particularly, studies have observed significant associations between quetiapine and metabolic and endocrine system (hyperglycemia, galactorrhea, dyslipidemia, etc.) and cardiovascular (cardiomyopathy, myocarditis, tachycardia, hypotension, prolonged QT interval, etc.) symptoms, although more serious consequences, including death, have been reported.^3^, ^4^, ^5^, ^6^

Evidence is accumulating from the reports and mechanism studies to support a possible association between quetiapine and cardiotoxicity. A case‐crossover study using a nationwide population‐based sample obtained from Taiwan's National Health Insurance Research Database reported that antipsychotic drug use such as quetiapine was associated with a 1.53‐fold increased risk of ventricular arrhythmia and/or sudden cardiac death.^7^ According to the International Drug Monitoring Program of the World Health Organization, quetiapine‐induced myocarditis and cardiomyopathy have become the common manifestations in pathology.^8^ Berge et al^6^ reported that treatment with low‐dose quetiapine/olanzapine for 6–12 months was significantly associated with an increased risk of cardiometabolic mortality (adjusted HR 1.89; 95% CI, 1.22–2.92, *p* = 0.004). Both researches by Wenzel‐Seifert et al^9^ and Hasnain et al^10^ observed that torsade de pointes (TdP) was in association with the use of newer antipsychotic drugs, which mainly included quetiapine. The widely reported cardiovascular side effects have also raised concerns of the Danish Medicine Agency or the European Medicine Agency, which has therefore been cautious about expanding the use of quetiapine.^11^, ^12^ In particular, the FDA have warned in the instructions that elderly patients with dementia‐related psychosis was associated with an increased risk of death from cardiovascular diseases (e.g., heart failure, sudden death) and infections (e.g., pneumonia) when treated with quetiapine.

Due to the limitations of clinical trials, such as specific population, relatively small sample size, limited duration of follow‐ups, and strict inclusion and exclusion criteria, the association between quetiapine and rare cardiac AEs is still unclear, suggesting continuous post‐marketing surveillance is urgent.^13^ The long‐term safety of drugs and the occurrence of rare or serious AEs are largely assessed using post‐marketing surveillance data, which increases the value of spontaneous reporting systems such as Food and Drug Adverse Event Reporting System (FAERS).^14^ The FAERS database is now widely used to identify pharmacovigilance risk signals in real‐world clinical settings.^15^ The purpose of this study was to utilize standardized information from the FAERS, to describe the characteristics of quetiapine‐related cardiac AE reports from stratification analysis, clinical priority of signals, time‐to‐onset, and the serious outcomes.

## 2 METHODS

### 2.1 Study design and data sources

A disproportionality analysis, designed as a case/non‐case study, was used to quantify the association between quetiapine and cardiological AEs. It calculated the proportion of occurring target AEs between a target drug (case) and all other drugs (non‐case).^16^ A significant safety signal occurs when a target drug is reported more frequently to induce a target AE than all other drugs. The data were sourced from the FAERS Quarterly Data Extract Files, available at https://fis.fda.gov/extensions/FPD‐QDE‐FAERS/FPD‐QDE‐FAERS.html. To get the latest reports, all reports recorded in FAERS covering the period between the first quarter (Q1) of 2018 and the 2022 Q1, were extracted in our study.

### 2.2 Data extraction and descriptive analysis

The database included seven data files, namely patient demographic information (DEMO), drug/biologic information (DRUG), adverse events (REAC), patient outcomes (OUTC), report sources (RPSR), start/end dates of drug therapy (THER), and indications for drug (INDI).^14^ In the FAERS database architecture, a relation was built to connect each data file by some special identification numbers (such as caseid, primaryid). Moreover, the cases deleted by FDA or manufacturers for various reasons were collected in Deleted files. Because multiple versions of a report would be reported, the deduplication process should be performed before statistical analysis, to ensure the uniqueness of the report.^17^ The caseid and the primaryid were used as the key filters in our study to remove duplicate records. First, the reports in the Deleted files were deleted. Then, the report with the higher primaryid was selected when the caseid was the same. We further removed reports where the data were not available, when we counted patient information (e.g., sex and age). We extracted reports using generic name (quetiapine in drugname and prod_ai columns) and trade name (SEROQUEL in drugname column) in the DRUG file. To improve the reported association between drug and AEs, only the role_cod as primary suspected (PS) was selected. AEs in FAERS were coded using the preferred term (PT) according to standardized Medical Dictionary for Regulatory Activities (MedDRA 25.0), which were divided into five levels: system organ class (SOC), high level group term (HLGT), high level term (HLT), preferred term (PT), and lowest level term (LLT).^18^ The quetiapine‐associated AEs extracted from the REAC files, were used to calculate the frequency and intensity at PT and SOC levels. All the PTs under the SOC of cardiac disorders (SOC: 10007541) in the MedDRA 25.0 were calculated in our study. Further, because a PT can be normally affiliated to more than one SOC in MedDRA 25.0, the Aes in reports were classified to the corresponding PT and SOC levels. All data processing was performed using MYSQL 8.0 (Oracle, Sweden), Navicat Premium 15, Microsoft EXCEL 2019, and the GraphPad Prism 8 (GraphPad Software, CA, USA).

**FIGURE 1:**
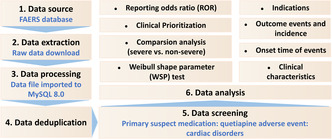
Multistep process of data extraction, processing, and analysis from Food and Drug Administration adverse event reporting system (FAERS) database.

Subsequently, descriptive analyses were carried out to summarize the clinical characteristics of all quetiapine‐associated reports. Detailed information will be calculated if the data are available, such as gender, age, weight, reporting countries, indications, outcomes, and time‐to‐onset, etc. A flowchart including the multistep process of data extraction, processing, and analysis is shown in Figure 1.

### 2.3 Statistical analysis

Based on the disproportionality analysis using a 2 × 2 table (Table S1), the reporting odds ratio (ROR) was employed to identify an association between a drug and an AE. All cardiological AE reports were selected to perform signal strength of reports of quetiapine at both PT (cardiological AEs) and SOC (cardiac disorders) levels in FAERS database. To reduce the likelihood of false positives, the threshold was set, and a significant signal was defined when the lower limit of the ROR 95% confidence interval (CI) exceeded 1 with at least five reports.^19^

The outcomes of AE reports were classified as serious or nonserious in FAERS. The serious outcomes included death (DE), life‐threatening (LT), inpatient hospitalization or prolongation of existing hospitalization (HO), disability (DS), congenital anomaly (CA), required intervention to prevent permanent impairment/damage (RI), as well as other serious/important medical event (OT). Some reports might list more than one specific outcome (e.g., one report experienced DS, LT, and then DE), which recorded in the OUTC file. However, the serious and nonserious reports were also compared to clarify the severity of the detected safety signals and identify risk factors (gender, age, weight, and types of AEs) in patients. Proportions were compared using a Pearson's chi‐squared (χ^2^) or Fisher's exact test, and Mann–Whitney U test was applied for continuous non‐normal distribution data, such as age and weight. Data were analyzed using SPSS (v22.0; IBM Corp., Armonk, NY, United States), and statistical significance was set at a two‐tailed *p* < 0.05. To further explore the influence of different stratification regimens on the correlation between quetiapine and cardiac disorders, we performed subgroup analysis by gender (female and male), age (<18, 18 ≤ and ≤ 65, >65 years), weight (<80, 80 ≤ and ≤ 100, and >100 kg), and reporters (health‐care professional and consumer) separately.

### 2.4 Clinical prioritization of signals

AEs with significant disproportionality were scored and ranked according to clinical priority in our study. A semiquantitative score was performed to prioritize disproportionality signals by assessing five different features, including number of AE reports, the lower limit of the ROR 95% CI (ROR_025_) values, proportion of death, characterization as designated medical events (DMEs) or important medical events (IMEs), and evidence evaluation.^19^, ^20^ According to three levels of clinical importance, a composite score between 0–4, 5–7, or 8–10 was identified as AEs with weak, moderate, or strong clinical priority, respectively. The detailed information is shown in Table S2.

### 2.5 Time‐to‐onset analysis and sensitivity analysis

Time‐to‐onset (TTO) was calculated as the duration between the occurrence date of AEs (EVENT_DT in DEMO file) and start date of quetiapine use (START_DT in THER file).^13^ Only the reports with available TTO data were analyzed, and reports with input errors (EVENT_DT earlier than START_DT) were excluded before analysis, to ensure the accuracy of calculation. The incidence of AEs often varies over time, and the Weibull shape parameter (WSP) test is used for statistical analysis of TTO to describe the risk that the incidence of AEs increases or decreases over time.^21^, ^22^, ^23^ The shape of Weibull distribution was described by two parameters: scale (α) and shape (β). In the analysis of WSP test, the shape parameter β of the Weibull distribution will be considered and discussed to forecast the hazard without a reference population. However, three hazard types are described in WSP test: early failure type means the hazard of the AEs decreases over time (β < 1 and 95% CI < 1); random failure type means the hazard of the AEs constantly occurs over time (β was equal to or nearly 1 and its 95% CI included the value 1); wear‐out failure type means the hazard of the AEs increases over time (β > 1 and 95% CI > 1).

In order to predict the risk of increase or decrease of these quetiapine‐associated cardiological AEs over time, we calculated the median TTO and WSP of signals with moderate or weak clinical priority after quetiapine use. The WSP tests were performed using Minitab statistical software (v20.0; Minitab LLC).

Moreover, we also considered the effect of concomitant drugs on quetiapine‐related cardiac AEs, and concomitant therapy was defined as quetiapine being the “primary suspect.” and other drugs were listed as “second suspect,” “concomitant,” or “interacting.” Subsequently, the sensitivity analysis was performed after the exclusion of the reports associated with potential suspicious drugs.^24^

## 3 RESULTS

### 3.1 Descriptive analysis

From January 2018 to March 2022, the FAERS database has recorded 6,437,182 AE reports after exclusion of duplicates, including 1415 quetiapine‐associated cardiac AEs in 1004 patients. The clinical characteristics of quetiapine‐induced AEs are described in Table 1. Among all reports, females accounted for a larger proportion than males (61.89% vs 38.11%). Patients were mainly aged 18–65 years (72.60%) and >65 years (19.82%), with a median age of 44.03 (IQR 28–59) years. Serious outcomes of cardiac and overall AE reports, including 143 (16.49%) and 757 (16.19%) deaths, were recorded in 867 and 4677 cases, respectively. Additionally, 21.61% of the patients reported onset time of the cardiac AEs, with the median time‐to‐onset of 1 day (IQR 0–30 days). The most reported indication of quetiapine was psychiatric disorders (*n* = 443, 90.22%). Most reports were submitted by health‐care professionals (*n* = 654, 70.70%) and consumers (*n* = 271, 29.30%), respectively. The country with the most cardiac AE reports was USA (*n* = 297, 29.58%), followed by United Kingdom (*n* = 164, 16.33%) and Canada (*n* = 105, 10.46%).

**TABLE 1:** Clinical characteristics of patients with quetiapine‐associated cardiac adverse events.

Characteristics	Quetiapine induced cardiac AEs (*n* = 1004)		Quetiapine induced overall AEs (*n* = 6170)	
	Available number	Value	Available number	Value
Gender, *n* (%)	929 (92.53%)	‐	5709 (92.53%)	‐
Female	‐	575 (61.89%)	‐	3356 (58.78%)
Male	‐	354 (38.11%)	‐	2353 (41.22%)
Age (years), *n* (%)	792 (78.88%)	‐	4728 (76.63%)	‐
<18	‐	60 (7.58%)	‐	345 (7.30%)
18 ≤ and ≤ 65	‐	575 (72.60%)	‐	3403 (71.98%)
>65	‐	157 (19.82%)	‐	980 (20.72%)
Median (IQR)	‐	44.03 (28–59)	‐	45 (31–61)
Weight (Kg), *n* (%)	278 (27.69%)	‐	1350 (21.88%)	‐
<80	‐	167 (60.07%)	‐	864 (64.00%)
80 ≤ and ≤ 100	‐	72 (25.90%)	‐	322 (23.85%)
>100	‐	39 (14.03%)	‐	164 (12.15%)
Median (IQR)	‐	70.55 (56.34–90.70)	‐	70.30 (58.50–88.34)
Reported countries, *n* (%)	1004 (100%)	‐	6170 (100%)	‐
America (USA)	‐	297 (29.58%)	‐	1936 (31.38%)
United Kingdom (UK)		164 (16.33%)		765 (12.40%)
Italy (IT)		61 (6.08%)		577 (9.35%)
Germany (DE)		100 (9.96%)		514 (8.33%)
Canada (CA)		105 (10.46%)		503 (8.15%)
Others	‐	277 (27.59%)	‐	1875 (30.39%)
Indications, *n* (%)	491 (48.90%)	‐	3147 (51.00%)	‐
Psychiatric disorders	‐	443 (90.22%)	‐	2835 (90.09%)
Others	‐	48 (9.78%)	‐	312 (9.91%)
Outcomes, *n* (%)	1004 (100%)	‐	6170 (100%)	‐
Nonserious outcome	‐	137 (13.65%)	‐	1493 (24.20%)
Serious outcome	‐	867 (86.35%)	‐	4677 (75.80%)
Death	‐	143 (16.49%)	‐	757 (16.19%)
Life‐threatening	‐	116 (13.38%)	‐	398 (8.51%)
Hospitalization	‐	397 (45.79%)	‐	1829 (39.11%)
Disability	‐	44 (5.07%)	‐	185 (3.96%)
Other serious outcomes	‐	636 (73.36%)	‐	3093 (66.13%)
Time‐to‐onset (days)	217 (21.61%)	‐	1271 (20.60%)	‐
Median (IQR)	‐	1 (0–30)	‐	1 (0–31)
Reporters, *n* (%)	925 (92.13%)	‐	5729 (92.86%)	‐
Health professional	‐	654 (70.70%)	‐	4271 (74.55%)
Consumer	‐	271 (29.30%)	‐	1458 (25.45%)
Reporting year, *n* (%)	1004 (100%)	‐	6170 (100%)	‐
2022 Q1*	‐	60 (5.98%)	‐	357 (5.79%)
2021	‐	231 (23.01%)	‐	1437 (23.29%)
2020	‐	202 (20.12%)	‐	1412 (22.88%)
2019	‐	252 (25.10%)	‐	1585 (25.69%)
2018	‐	259 (25.80%)	‐	1379 (22.35%)

Abbreviations: AEs: Adverse events; *n*: number of cases.

^*^ The first quarter of 2022.

### 3.2 Disproportionality analysis

**FIGURE 2:**
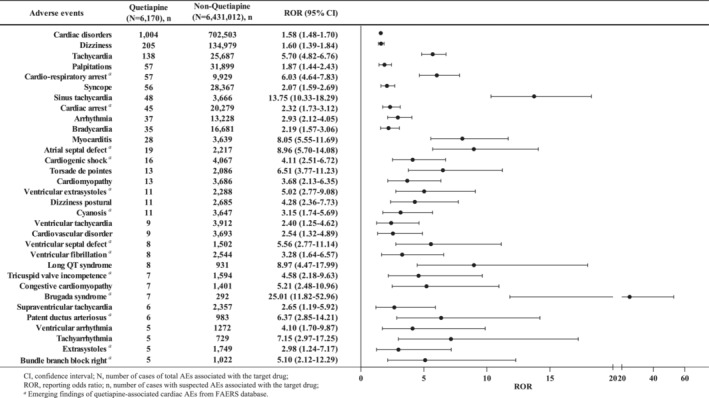
Reporting odds ratios (ROR) with 95% CI for all positive quetiapine‐associated cardiac adverse events with at least five counts.

During the study period, a total of 31 different PTs of quetiapine‐associated cardiac AEs were identified in FAERS database in at least five cases (Figure 2). The most commonly reported cardiac AEs were dizziness (*n* = 205), tachycardia (*n* = 138), palpitations (*n* = 57), and cardiorespiratory arrest (*n* = 57). Figure 2 presented a full list of disproportionality analysis results. In the entire database, quetiapine‐related cardiac disorders were reported significantly more frequently than non‐quetiapine, with an ROR_025_ of 1.48. Further, investigation of cardiac AE signals revealed that 31 cardiac AEs showed statistically significant signal strengths after quetiapine treatment compared with non‐quetiapine‐related cardiac AEs, with values of signals ranging from a ROR_025_ of 1.19 (supraventricular tachycardia) to 52.96 (Brugada syndrome). Other quetiapine‐related nonpositive cardiac AE signals are shown in Table S3. Results of signal strength of quetiapine at the SOC level in Table S4 demonstrated that there were 12 SOCs exhibiting remarkable association with quetiapine treatment, among which including cardiac disorders with the ROR of 1.58 (1.48–1.70).

### 3.3 Serious vs. Nonserious cases

Data in Table 2 showed that there were no statistically significant differences in sex (χ^2^ = 1.395, *p* = 0.237), age (43 vs 51 years; *p* = 0.093), and body weight (71 vs 70 kg; *p* = 0.476) between severe and non‐severe cases of cardiac AE patients receiving quetiapine. Dizziness (χ^2^ = 13.362, *p* < 0.001), cardiorespiratory arrest (χ^2^ = 9.549, *p* = 0.002), and palpitations (χ^2^ = 10.671, *p* = 0.001) were more likely to be reported as serious AEs, while other 28 AEs were more tended to be reported as nonserious AEs with *p* > 0.05, such as tachycardia (χ^2^ = 0.239, *p* = 0.625), arrhythmia (χ^2^ = 1.000, *p* = 0.317), and myocarditis (*p* = 0.161), etc. Of note, all AEs outcomes for cardiorespiratory arrest (*n* = 57), atrial septal defect (*n* = 19), torsade de pointes (*n* = 13), ventricular extrasystoles (*n* = 11), ventricular tachycardia (*n* = 9), ventricular septal defect (*n* = 8), ventricular fibrillation (*n* = 8), Brugada syndrome (*n* = 7), ventricular arrhythmia (*n* = 5), and tachyarrhythmia (*n* = 5) were severe cases.

**TABLE 2:** Differences in clinical characteristics of severe and non‐severe reports.

	Serious cases (*n* = 867)	Nonserious cases (*n* = 137)	Statistics	*p* value
Gender, *n* (%)				
Female	503 (87.48%)	72 (12.52%)	1.395^a^	0.237^b^
Male	300 (84.75%)	54 (15.25%)	‐	‐
Age, years (Median)	43	51	−1.679^c^	0.093^d^
Weight, Kg (Median)	71	70	−0.713^c^	0.476^d^
Types of AEs, *n* (%)	‐	‐	‐	‐
Dizziness	161	44	13.362^a^	< 0.001^b^
Tachycardia	121	17	0.239^a^	0.625^b^
Cardiorespiratory arrest^e^	57	0	9.549^a^	0.002^b^
Syncope	52	4	2.128^a^	0.145^b^
Cardiac arrest^e^	43	2	3.385^a^	0.066^b^
Palpitations	41	16	10.671^a^	0.001^b^
Sinus tachycardia	37	11	3.677^a^	0.055^b^
Arrhythmia	34	3	1.000^a^	0.317^b^
Bradycardia	31	4	‐	1.000^f^
Myocarditis	27	1	‐	0.161^f^
Atrial septal defect^e^	19	0	‐	0.094^f^
Cardiogenic shock^e^	15	1	‐	0.711^f^
Torsade de pointes	13	0	‐	0.235^f^
Ventricular extrasystoles^e^	11	0	‐	0.378^f^
Cardiomyopathy	11	2	‐	0.694^f^
Dizziness postural	10	1	‐	1.000^f^
Ventricular tachycardia	9	0	‐	0.619^f^
Cyanosis^e^	9	2	‐	0.653^f^
Cardiovascular disorder	9	0	‐	0.619^f^
Ventricular septal defect^e^	8	0	‐	0.608^f^
Ventricular fibrillation^e^	8	0	‐	0.608^f^
Long QT syndrome	7	1	‐	1.000^f^
Brugada syndrome^e^	7	0	‐	0.602^f^
Tricuspid valve incompetence^e^	6	1	‐	1.000^f^
Patent ductus arteriosus^e^	6	0	‐	1.000^f^
Congestive cardiomyopathy	6	1	‐	1.000^f^
Ventricular arrhythmia	5	0	‐	1.000^f^
Tachyarrhythmia	5	0	‐	1.000^f^
Supraventricular tachycardia	4	2	‐	0.192^f^
Extrasystoles^e^	4	1	‐	0.521^f^
Bundle branch block right^e^	4	1	‐	0.521^f^

*Note*: The AEs listed above were AEs with significant signal strengths. *p* ˂ 0.05 were considered statistically significant.

^a^ The chi‐square (χ^2^) statistic of the Pearson chi‐square test.

^b^ Proportions were compared using Pearson chi‐square test.

^c^ The *Z* statistic of the Mann–Whitney U test.

^d^ Mann–Whitney U test.

^e^ Emerging findings of quetiapine‐associated cardiac AEs from FAERS database.

^f^ Fisher's exact test.

### 3.4 Subgroup analysis

**FIGURE 3:**
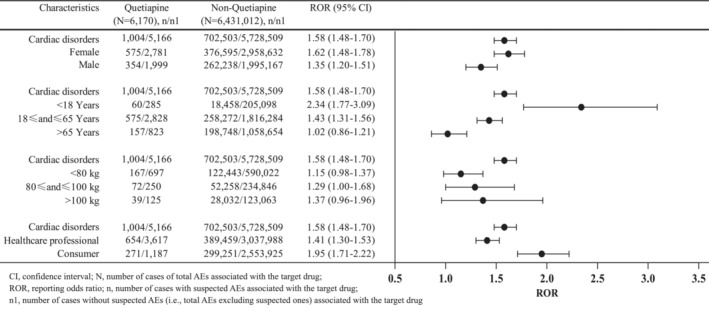
Subgroup analysis of quetiapine‐related cardiac disorders.

As shown in Figure 3, cardiac disorders were separately assessed stratifying by sex, age, weight, and type of reporters. Results demonstrated that the lower limits of ROR values were >1 when stratified by sex and reporters' type, indicating there was still a strong statistical correlation between quetiapine and cardiac disorders. In females, ROR_025_ was found to be 1.48, whereas it was 1.20 in males, indicating a significant association of quetiapine with cardiac AEs in both male and females. Moreover, it suggested that women were more likely to experience cardiotoxicity with quetiapine than men. The majority of quetiapine‐associated cardiac AEs cases were found in the 18–65 years age groups. However, it did not show significant association between cardiac disorders and quetiapine treatment when stratified by age and body weight, primarily in subgroups of patients >65 years old and body weight <80 kg and >100 kg.

### 3.5 Clinical prioritization of the disproportionality signals

In total, 14 of the 31 AEs (45.16%) with statistically significant disproportionality signals were categorized as IMEs, whereas only two represented DMEs (6.45%), including torsade de pointes and ventricular fibrillation (Table 3). According to the clinical priority assessment, 22 (70.97%), nine (29.03%), and zero cardiac AEs were identified as weak, moderate, and strong clinical priority, respectively. Cardiorespiratory arrest (*n* = 57, ROR_025_ = 4.64) and myocarditis (*n* = 28, ROR_025_ = 5.55) were graded as moderate clinical priority with the highest priority scores of 7. Besides, 18 AEs were evaluated as strong clinical evidences with “++.”

**TABLE 3:** Clinical priority assessing results of disproportionality signals.

PTs	*n*	ROR_025_	Death (*n*)	IMEs‐DMEs	Relevant evidence evaluation	Priority level (score)
Dizziness	205	1.39	8	NA	++	Weak (4)
Tachycardia	138	4.82	3	IME	++	Moderate (6)
Cardiorespiratory arrest^a^	57	4.64	57	IME	+	Moderate (7)
Palpitations	57	1.44	0	IME	++	Moderate (5)
Syncope	56	1.59	0	NA	++	Weak (4)
Sinus tachycardia	48	10.33	0	IME	++	Moderate (6)
Cardiac arrest^a^	45	1.73	25	IME	+	Moderate (5)
Arrhythmia	37	2.12	11	IME	++	Moderate (6)
Bradycardia	35	1.57	6	IME	++	Weak (4)
Myocarditis	28	5.55	9	IME	++	Moderate (7)
Atrial septal defect^a^	19	5.7	0	NA	−	Weak (3)
Cardiogenic shock^a^	16	2.51	1	NA	−	Weak (2)
Torsade de pointes	13	3.77	0	DME	++	Moderate (6)
Cardiomyopathy	13	2.13	1	IME	++	Moderate (5)
Dizziness postural	11	2.36	0	NA	++	Weak (4)
Ventricular extrasystoles^a^	11	2.77	0	NA	−	Weak (2)
Cyanosis^a^	11	1.74	0	NA	−	Weak (1)
Cardiovascular disorder	9	1.32	3	NA	++	Weak (3)
Ventricular tachycardia	9	1.25	1	IME	++	Weak (3)
Long QT syndrome	8	4.47	0	NA	++	Weak (3)
Ventricular fibrillation^a^	8	1.64	0	DME	−	Weak (2)
Ventricular septal defect^a^	8	2.77	0	NA	−	Weak (1)
Congestive cardiomyopathy	7	2.48	0	IME	++	Weak (4)
Brugada syndrome^a^	7	11.82	0	NA	−	Weak (2)
Tricuspid valve incompetence^a^	7	2.18	0	NA	−	Weak (1)
Supraventricular tachycardia	6	1.19	0	IME	++	Weak (3)
Patent ductus arteriosus^a^	6	2.85	0	NA	−	Weak (1)
Ventricular arrhythmia	5	1.7	2	IME	++	Weak (4)
Tachyarrhythmia	5	2.97	0	IME	++	Weak (4)
Bundle branch block right^a^	5	2.12	0	NA	−	Weak (1)
Extrasystoles^a^	5	1.24	0	NA	−	Weak (0)

Abbreviations: NA, Not Applicable (for relevant criteria); *n*, number of cases; PTs, preferred Terms; ROR_025_, the lower limit of 95% confidence interval of ROR. A priority score between 8–10, 5–7, or 0–4 represents the signal with strong, moderate, or weak clinical priority, respectively.

^a^ Emerging finding of quetiapine‐associated cardiac AEs from FAERS database.

### 3.6 Time‐to‐onset analysis and sensitivity analysis

As shown in Table 4, the median onset time of moderate and weak AE signals associated with quetiapine was 0 (IQR 0–8) and 4 (IQR 0–59.5) days, respectively. In the WSP analysis, the upper limits of 95% CI of the shape parameters β were <1, suggesting these moderate and weak clinical priority signals had early failure types, and the risk of cardiac AEs occurrence gradually decreased over time.

**TABLE 4:** Results of time‐to‐onset analysis for signals with moderate/weak prioritization.

Prioritization	TTO (days)			Weibull distribution				Failure type
	Cases	‐		Scale parameter		Shape parameter		
	*n*	Median (IQR)	Min‐max	α	95% CI	β	95% CI	
Moderate	93	0 (0–8)	0–517	0.13	0.03–0.58	0.15	0.13–0.17	Early failure
Weak	94	4 (0–59.5)	0–520	4.75	1.50–15.04	0.18	0.15–0.22	Early failure

*Note*: *n*: number of cases with available time‐to‐onset; IQR: interquartile range; TTO: time‐to‐onset. When TTO is 0 days, the adverse event occurred within the same day with the therapy.

Among quetiapine‐induced cardiac AEs reports, there were 721 cases (71.81%) in combination with other drugs. The reports of quetiapine combined with one, two, and three drugs were recorded in 157 (15.64%), 124 (12.35%), and 97 (9.66%) cases, respectively. The top 20 concomitant drugs are shown in Table 5, and the top five were lorazepam (*n* = 88, 12.21%), sertraline hydrochloride (*n* = 75, 10.40%), mirtazapine (*n* = 65, 9.02%), aripiprazole (*n* = 64, 8.88%), and lamotrigine (*n* = 62, 8.60%). The sensitivity analysis was performed after exclusion of drugs (aripiprazole, risperidone, and olanzapine) that belonged to the second‐generation antipsychotics. Although the number of quetiapine‐related cardiac AE reports were decreased from 1004 to 868 (ROR 1.33; 95% CI 1.24–1.43) after removing the reports of suspicious drugs, it still had a significant association between quetiapine and cardiac disorders. Moreover, five PTs no longer showed significant signals, including long QT syndrome, supraventricular tachycardia, ventricular arrhythmia, extrasystoles, and bundle branch block right. The detailed changes are shown in Table S5.

**TABLE 5:** Top 20 concomitant drugs for quetiapine‐related cardiac AEs from FAERS databases.

Concomitant drugs (TOP 20)	*N* (%)
Lorazepam	88 (12.21)
Sertraline hydrochloride	75 (10.40)
Mirtazapine	65 (9.02)
Aripiprazole	64 (8.88)
Lamotrigine	62 (8.60)
Venlafaxine hydrochloride	59 (8.18)
Clonazepam	56 (7.77)
Aspirin	49 (6.80)
Risperidone	49 (6.80)
Diazepam	45 (6.24)
Olanzapine	45 (6.24)
Trazodone hydrochloride	45 (6.24)
Acetaminophen	43 (5.96)
Escitalopram oxalate	43 (5.96)
Gabapentin	39 (5.41)
Pregabalin	37 (5.13)
Levothyroxine sodium	33 (4.58)
Metformin hydrochloride	33 (4.58)
Zopiclone	32 (4.44)
Fluoxetine hydrochloride	31 (4.30)

*Note*: *N*, number of adverse event reports.

## 4 DISCUSSION

This pharmacovigilance study comprehensively and systematically provided the latest findings of quetiapine‐associated cardiac safety profiles by post‐marketing based on the FAERS database. Our results are in line with previous clinical trials and literature reviews highlighting the association between exposure to antipsychotics and cardiac AEs. According to a recent epidemiological study, Weeke et al^25^ found that quetiapine, as the only atypical antipsychotic drug, was associated with 3.64 times increased risk of out‐of‐hospital cardiac arrest. In a randomized double‐blind study by Nielsen et al,^26^ results revealed that there were 11 patients (9.6%) receiving quetiapine who experienced more than 20 ms QTcF prolongation. Hagiwara et al^27^ have reported a 37‐year‐old man who developed myocarditis after starting treatment with quetiapine for bipolar disorder. The cytokine release and catecholamine hyperactivation suggested to be associated with quetiapine administration may have been the etiological mechanism. However, studies on quetiapine‐induced cardiac AEs based on the large‐sample real‐world data were quite limited, and none of the studies provided data after stratifying by sex, age, and weight, strongly underling the need for constant post‐marketing surveillance.

Fortunately, we innovatively used multidimensional analytic approaches, such as subgroup analysis, clinical priority of signals and the serious outcomes, to provide insight into the primary results. In our study, a total of 1004 reports of quetiapine‐associated cardiac AEs were obtained, and 31 significant AEs with at least five reports were detected. The most frequently reported cardiac AEs of quetiapine were dizziness (*n* = 205, ROR = 1.60), tachycardia (*n* = 138, ROR = 5.70), palpitations (*n* = 57, ROR = 1.87), and cardiorespiratory arrest (*n* = 57, ROR = 6.03), which were consistent with clinical trials.^7^, ^28^ Among the 31 AEs, 13 AEs which were not reported in the drug label, were identified as new and unexpected signals, such as atrial septal defect, ventricular extrasystoles, and tricuspid valve incompetence, etc. The full list of all new quetiapine‐associated cardiac AEs is presented in Figure 2.

This study suggests that there are significant differences (*p* < 0.05) in three AEs among the 31 cardiac AEs, when compared serious cases with nonserious cases. Patients gender (*p* = 0.237), age (*p* = 0.093), and weight (*p* = 0.476) may not be associated with increased risk of quetiapine‐related cardiac AEs severity. However, the descriptive analysis (Table 1) showed that females (61.89%) were more likely to report cardiac AEs than males (38.11%), in agreement with a study based on FAERS that females (58.4%) had a higher proportion of cardiac AEs than males (41.6%) after antipsychotics treatment.^29^ Further comparison of severe and non‐severe cases demonstrated that the proportion of females with serious cardiac AEs was numerically higher than that of males (87.48% vs 84.75%), but there was no statistically significant difference between the two groups. Results were consistent with previous studies that women were at a higher risk of reporting severe cardiac AEs after receiving antipsychotics.^30^, ^31^, ^32^ Both studies by Harrigan et al^31^ and Roe et al^32^ found that although women were at a lower risk of sudden cardiac death, they had a higher risk of induced long QT syndrome from antipsychotic drugs. To date, sex differences in quetiapine‐induced AEs have not been well studied, but some AEs, such as weight gain, elevated lipids, and cardiac effects, have been reported to be particularly associated with women.^30^ The present subgroup analysis revealed that females were associated with more cardiac AEs than males with a higher ROR value (1.62 vs. 1.35). The mechanism of gender‐specific effects on quetiapine‐related cardiotoxicity may be related to hormones and pharmacokinetic properties.^33^

In our study, patients aged 18–65 years reported more frequently cardiac AEs (*n* = 575, 72.60%) than those aged >65 years (*n* = 157, 19.82%) and <18 years (*n* = 60, 7.58%), which were consistent with a FAERS study that the median patient age among quetiapine abuse‐related event reports was 44 years (IQR = 31–55).^34^ On the contrary, younger patients generally had numerically higher ROR estimates than older adults (2.34 vs. 1.43) in the subgroup analysis (Figure 2), suggesting youths taking quetiapine might be more likely to have cardiac AEs. Moreover, severe cases were also reported at younger ages than non‐severe cases (median age 43 vs 51 years), but there was no statistical difference among the two groups (*p* = 0.093). However, Yunusa et al^35^ reported the opposite results that antipsychotics were associated with potentially serious AEs in vulnerable older adults with higher RORs of hospitalization due to age‐related reduction in the ability to metabolize and excrete drugs, thus leading to a higher plasma concentration of quetiapine. Another study also demonstrated an increased risk of cardiovascular events in older patients with antipsychotic drugs, including quetiapine.^36^ Therefore, cautions are necessary when prescribing quetiapine to older women and children.

We conducted subgroup analysis by body weight (<80, 80–100, and >100 kg), and found that only the subgroup of body weight 80–100 kg presented significant signal strength with ROR_025_ = 1. The other two groups (<80 and >100 kg) did not show an association between quetiapine and cardiac AEs because of ROR_025_ < 1. A Bayesian meta‐analysis of 41 short‐term trials of second‐generation antipsychotics found a significant mean weight gain (1.74 kg; 95% CI, 0.99–2.50) during treatment with quetiapine.^37^ Jensen et al^38^ also reported that quetiapine was associated with significantly greater weight gain and adverse changes in cardiometabolic outcomes in youths. Results suggested body weight might, in part, be associated with quetiapine‐induced cardiac AEs.

Clinical prioritization assessment was employed to prioritize quetiapine‐related cardiac safety signals. Our results showed that zero strong, nine moderate, and 22 weak clinical priority signals were identified. The strongest priority signals were cardiorespiratory arrest and myocarditis with score 7. In addition, the most frequently reported quetiapine‐associated priority cardiac AEs were tachycardia (*n* = 138, score 6), palpitations (*n* = 57, score 5), sinus tachycardia (*n* = 48, score 6), arrhythmia (*n* = 37, score 6), torsade de pointes (*n* = 13, score 6), and cardiomyopathy (*n* = 13, score 5), which were consistent with the drug label. Of note, 12 of the 31 AEs were serious cases (Table 2). Quetiapine‐related cardiac arrest was more likely to be reported as a severe AE. Wu et al^7^ reported a significantly increased risk of ventricular arrhythmia and/or sudden cardiac death for quetiapine (adjusted OR = 1.29; 95% CI, 1.07–1.56). According to data from Danish Cardiac Arrest Register (2001–2010), 2205 (7.6%) of 28,947 out‐of‐hospital cardiac arrest patients received antipsychotics treatment at the time of the event, and quetiapine was identified to be associated with cardiac arrest (OR = 3.64; 95% CI, 1.59–8.30).^25^ Other large epidemiological studies have also found a dose‐dependent relationship between quetiapine and sudden cardiac death.^4^, ^39^ Particularly, in our study, a total of 57 and 45 cases of quetiapine‐associated cardiorespiratory arrest and cardiac arrest were obtained from FAERS database, and there were 57 (100.00%) and 25 (55.56%) death, respectively (Table 3), which were corresponding to previous reports and emerging data from the World Health Organization pharmacovigilance database (VigiBase) and FAERS.^40^, ^41^ Thus, physicians should perform baseline measurements of cardiovascular‐related indicators before prescription to maximize patient benefit.

In the TTO analysis (Table 4), the median TTO for moderate and weak clinical priority signals were 0 and 4 days, respectively, revealing quetiapine‐related moderate signals often occurred on the day of treatment. Furthermore, all of the cardiac AEs had early failure type characteristics, suggesting that most of the patients developed cardiac AEs in a few days with quetiapine treatment, and that the risk of cardiac AEs occurrence would be gradually decreased over time. Hence, clinicians should be aware of the potential toxicity of quetiapine and that cardiac AEs may occur on the day of treatment, particularly when treating patients sharing risk factors with females and the older population. To correctly grasp the balance between the efficacy and safety of quetiapine, timely and effective treatment of cardiac AEs, and helping patients to maintain long‐term medication, prospective studies and long‐term follow‐ups are needed to verify our results.

The exact mechanisms underlying quetiapine‐induced cardiac injury are still far to be completely understood. Toxicological studies have shown that quetiapine promoted the necroptotic cell death to induce cardiac toxicity, and selective CB1R antagonists or CB2R agonists might provide beneficial potentials against quetiapine‐induced cardiotoxicity.^8^ Recently, a number of preclinical studies have proposed the potential role of pro‐inflammatory cytokines, catecholamines, and oxidative stress in antipsychotic‐induced cardiotoxicity.^1^ Furthermore, concomitant medications should also be considered because the use of other antipsychotics or neurologic agents has been reported to significantly increase the risk of cardiac AEs. In our analysis, lorazepam, sertraline hydrochloride, mirtazapine, aripiprazole, and lamotrigine were the top five concomitant drugs of quetiapine. Recently, both the manufacturer of sertraline hydrochloride and the US FDA have warned the drug‐induced QTc interval prolongation and torsade de pointes when using sertraline hydrochloride.^42^ Besides, a study have demonstrated that lamotrigine toxicity manifested with minor‐moderate neurologic and/or electrocardiographic effects.^43^ Therefore, we cannot exclude the possibility that quetiapine combined with other drugs may increase the risk of cardiac AEs.

There are some limitations inherently shared by all pharmacovigilance databases. First, due to the nature of spontaneous reporting mechanism of the FAERS database (e.g., false, overreported, inaccurate, incomplete, and delayed reports), our analysis is subject to inevitable and unquantifiable biases. Second, because the total number of patients using quetiapine is not available in FAERS, we cannot calculate AE incidence and establish causality. Third, we focus only on AEs in cardiac disorders, and the deep relationship between quetiapine and other systemic organ classes remain unknown. Notwithstanding the above limitations, we systematically and comprehensively reveal the association between quetiapine and cardiotoxicity through an extensive analysis of a real‐world, large‐sample FAERS database, and prioritize cardiac AE signals, which provides valuable evidence for healthcare professionals to mitigate the risk of quetiapine‐associated cardiac AEs.

## 5 CONCLUSION

Our pharmacovigilance study systematically explored and quantified the potential cardiotoxicity induced by quetiapine, which obtained novel safety information about quetiapine. Among the 31 cardiac AEs, 13 new and unexpected AEs signals are confirmed. Besides, nine and 22 cardiac AEs were identified as moderate and weak clinical priority. The median TTO for moderate and weak clinical priority signals were 0 and 4 days, respectively, and all of the cardiac AEs had early failure type characteristics. The characteristics of cardiotoxicity spectrum found in this study are helpful for clinicians to recognize different cardiotoxicity and guide clinical practice. Some cardiac AEs have not received enough attention, and more cardiac examinations are recommended for high‐risk patients.

## AUTHOR CONTRIBUTIONS

Qilin Zhang and Yamin Shu contributed to conception and study design, and took responsibility for the collection, integrity, and accuracy of the data. All authors drafted the article, participated in data analyses and interpretation, and revisions of the article, and approved the final version.

## FUNDING INFORMATION

This study was supported by grants from National Natural Science Foundation of China (No. 82104476).

## CONFLICT OF INTEREST STATEMENT

The authors declare that they have no conflicts of interest.

## Supporting information


Data S1.

## Data Availability

The data generated during and/or analyzed during this study are available from the corresponding author upon reasonable request.

## References

[1] D'Errico S , Russa R , Maiese A , et al. Atypical antipsychotics and oxidative cardiotoxicity: review of literature and future perspectives to prevent sudden cardiac death. J Geriatr Cardiol. 2021;18(8):663‐685. 34527032 10.11909/j.issn.1671-5411.2021.08.002 PMC8390928

[2] Gummin DD , Mowry JB , Spyker DA , et al. 2018 annual report of the American Association of Poison Control Centers' National Poison Data System (NPDS): 36th annual report. Clin Toxicol (Phila). 2019;57(12):1220‐1413. 31752545 10.1080/15563650.2019.1677022

[3] De Hert M , Detraux J , van Winkel R , Yu W , Correll CU . Metabolic and cardiovascular adverse effects associated with antipsychotic drugs. Nat Rev Endocrinol. 2011;8(2):114‐126. 22009159 10.1038/nrendo.2011.156

[4] Ray WA , Chung CP , Murray KT , Hall K , Stein CM . Atypical antipsychotic drugs and the risk of sudden cardiac death. N Engl J Med. 2009;360(3):225‐235. 19144938 10.1056/NEJMoa0806994 PMC2713724

[5] Wu CS , Wu KY , Lo YR , et al. Psychotropic use and risk of stroke among patients with bipolar disorders: 10‐year nationwide population based study. J Affect Disord. 2018;226:77‐84. 28964996 10.1016/j.jad.2017.09.020

[6] Berge J , Abri P , Andell P , Movahed P , Ragazan DC . Associations between off‐label low‐dose olanzapine or quetiapine and cardiometabolic mortality. J Psychiatr Res. 2022;149:352‐358. 34785037 10.1016/j.jpsychires.2021.11.023

[7] Wu CS , Tsai YT , Tsai HJ . Antipsychotic drugs and the risk of ventricular arrhythmia and/or sudden cardiac death: a nation‐wide case‐crossover study. J Am Heart Assoc. 2015;4(2):e001568. 25713294 10.1161/JAHA.114.001568 PMC4345877

[8] Li X , Peng Z , Zhou Y , et al. Quetiapine induces myocardial necroptotic cell death through bidirectional regulation of cannabinoid receptors. Toxicol Lett. 2019;313:77‐90. 31220554 10.1016/j.toxlet.2019.06.005

[9] Wenzel‐Seifert K , Wittmann M , Haen E . QTc prolongation by psychotropic drugs and the risk of torsade de pointes. Dtsch Arztebl Int. 2011;108(41):687‐693. 22114630 10.3238/arztebl.2011.0687 PMC3221427

[10] Hasnain M , Vieweg WV . QTc interval prolongation and torsade de pointes associated with second‐generation antipsychotics and antidepressants: a comprehensive review. CNS Drugs. 2014;28(10):887‐920. 25168784 10.1007/s40263-014-0196-9

[11] Jakobsen KD , Wallach‐Kildemoes H , Bruhn CH , et al. Adverse events in children and adolescents treated with quetiapine: an analysis of adverse drug reaction reports from the Danish medicines agency database. Int Clin Psychopharmacol. 2017;32(2):103‐106. 27685179 10.1097/YIC.0000000000000148

[12] Lee SH , Kim HR , Han RX , Oqani RK , Jin DI . Cardiovascular risk assessment of atypical antipsychotic drugs in a zebrafish model. J Appl Toxicol. 2013;33(6):466‐470. 22120642 10.1002/jat.1768

[13] Chen C , Wu B , Zhang C , Xu T . Immune‐related adverse events associated with immune checkpoint inhibitors: an updated comprehensive disproportionality analysis of the FDA adverse event reporting system. Int Immunopharmacol. 2021;95:107498. 33725634 10.1016/j.intimp.2021.107498

[14] Shu Y , Ding Y , Liu Y , Wu P , He X , Zhang Q . Post‐marketing safety concerns with Secukinumab: a disproportionality analysis of the FDA adverse event reporting system. Front Pharmacol. 2022;13:862508. 35754494 10.3389/fphar.2022.862508 PMC9214234

[15] Shu Y , Ding Y , Dai B , Zhang Q . A real‐world pharmacovigilance study of axitinib: data mining of the public version of FDA adverse event reporting system. Expert Opin Drug Saf. 2022;21(4):563‐572. 34918584 10.1080/14740338.2022.2016696

[16] Peng L , Xiao K , Ottaviani S , Stebbing J , Wang YJ . A real‐world disproportionality analysis of FDA adverse event reporting system (FAERS) events for baricitinib. Expert Opin Drug Saf. 2020;19(11):1505‐1511. 32693646 10.1080/14740338.2020.1799975

[17] Shu Y , He X , Liu Y , Wu P , Zhang Q . A real‐world disproportionality analysis of Olaparib: data Mining of the Public Version of FDA adverse event reporting system. Clin Epidemiol. 2022;14:789‐802. 35789689 10.2147/CLEP.S365513 PMC9250344

[18] Shu Y , Ding Y , He X , Liu Y , Wu P , Zhang Q . Hematological toxicities in PARP inhibitors: a real‐world study using FDA adverse event reporting system (FAERS) database. Cancer Med. 2023;12(3):3365‐3375. 35871395 10.1002/cam4.5062 PMC9939145

[19] Gatti M , Antonazzo IC , Diemberger I , De Ponti F , Raschi E . Adverse events with sacubitril/valsartan in the real world: emerging signals to target preventive strategies from the FDA adverse event reporting system. Eur J Prev Cardiol. 2021;28(9):983‐989. 34402868 10.1177/2047487320915663

[20] Guo H , Wang B , Yuan S , et al. Neurological adverse events associated with Esketamine: a disproportionality analysis for signal detection leveraging the FDA adverse event reporting system. Front Pharmacol. 2022;13:849758. 35462924 10.3389/fphar.2022.849758 PMC9023790

[21] Sauzet O , Carvajal A , Escudero A , Molokhia M , Cornelius VR . Illustration of the weibull shape parameter signal detection tool using electronic healthcare record data. Drug Saf. 2013;36(10):995‐1006. 23673816 10.1007/s40264-013-0061-7

[22] Nakamura M , Umetsu R , Abe J , et al. Analysis of the time‐to‐onset of osteonecrosis of jaw with bisphosphonate treatment using the data from a spontaneous reporting system of adverse drug events. J Pharm Health Care Sci. 2015;1:34. 26819745 10.1186/s40780-015-0035-2 PMC4728763

[23] Abe J , Umetsu R , Mataki K , et al. Analysis of Stevens‐Johnson syndrome and toxic epidermal necrolysis using the Japanese adverse Drug event report database. J Pharm Health Care Sci. 2016;2:14. 27330825 10.1186/s40780-016-0048-5 PMC4915172

[24] Sharma A , Kumar A . Identification of novel signal of clobazam‐associated drug reaction with eosinophilia and systemic symptoms syndrome: a disproportionality analysis. Acta Neurol Scand. 2022;146(5):623‐627. 36029138 10.1111/ane.13690

[25] Weeke P , Jensen A , Folke F , et al. Antipsychotics and associated risk of out‐of‐hospital cardiac arrest. Clin Pharmacol Ther. 2014;96(4):490‐497. 24960522 10.1038/clpt.2014.139

[26] Nielsen J , Matz J , Mittoux A , et al. Cardiac effects of sertindole and quetiapine: analysis of ECGs from a randomized double‐blind study in patients with schizophrenia. Eur Neuropsychopharmacol. 2015;25(3):303‐311. 25583364 10.1016/j.euroneuro.2014.12.005

[27] Hagiwara H , Fukushima A , Iwano H , Anzai T . Refractory cardiac myocarditis associated with drug rash with eosinophilia and systemic symptoms syndrome due to anti‐bipolar disorder drugs: a case report. Eur Heart J ‐ Case Rep. 2018;2(4):yty100. 31020177 10.1093/ehjcr/yty100 PMC6426116

[28] Huang WL , Liao SC , Kuo TB , et al. The effects of antidepressants and quetiapine on heart rate variability. Pharmacopsychiatry. 2016;49(5):191‐198. 27023265 10.1055/s-0042-102964

[29] He L , Yu Y , Wei Y , Huang J , Shen Y , Li H . Characteristics and Spectrum of cardiotoxicity induced by various antipsychotics: a real‐world study from 2015 to 2020 based on FAERS. Front Pharmacol. 2021;12:815151. 35185550 10.3389/fphar.2021.815151 PMC8854762

[30] Aichhorn W , Whitworth AB , Weiss EM , Marksteiner J . Second‐generation antipsychotics: is there evidence for sex differences in pharmacokinetic and adverse effect profiles? Drug Saf. 2006;29(7):587‐598. 16808551 10.2165/00002018-200629070-00004

[31] Harrigan EP , Miceli JJ , Anziano R , et al. A randomized evaluation of the effects of six antipsychotic agents on QTc, in the absence and presence of metabolic inhibition. J Clin Psychopharmacol. 2004;24(1):62‐69. 14709949 10.1097/01.jcp.0000104913.75206.62

[32] Roe CM , Odell KW , Henderson RR . Concomitant use of antipsychotics and drugs that may prolong the QT interval. J Clin Psychopharmacol. 2003;23(2):197‐200. 12640222 10.1097/00004714-200304000-00013

[33] Seeman MV . The pharmacodynamics of antipsychotic drugs in women and men. Front Psych. 2021;12:650904. 10.3389/fpsyt.2021.650904 PMC8062799 33897500

[34] Evoy KE , Teng C , Encarnacion VG , et al. Comparison of quetiapine abuse and misuse reports to the FDA adverse event reporting system with other second‐generation antipsychotics. Subst Abuse: Res Treat. 2019;13:1178221819844205. 10.1177/1178221819844205 PMC6495438 31068753

[35] Yunusa I , Teng C , Karaye IM , Crounse E , Alsahali S , Maleki N . Comparative safety signal assessment of hospitalization associated with the use of atypical antipsychotics. Front Psych. 2022;13:917351. 10.3389/fpsyt.2022.917351 PMC9207238 35733796

[36] Gareri P , De Fazio P , De Fazio S , Marigliano N , Ferreri Ibbadu G , De Sarro G . Adverse effects of atypical antipsychotics in the elderly: a review. Drugs Aging. 2006;23(12):937‐956. 17154659 10.2165/00002512-200623120-00002

[37] Cohen D , Bonnot O , Bodeau N , Consoli A , Laurent C . Adverse effects of second‐generation antipsychotics in children and adolescents: a Bayesian meta‐analysis. J Clin Psychopharmacol. 2012;32(3):309‐316. 22544019 10.1097/JCP.0b013e3182549259

[38] Jensen KG , Correll CU , Rudå D , et al. Cardiometabolic adverse effects and its predictors in children and adolescents with first‐episode psychosis during treatment with quetiapine‐extended release versus aripiprazole: 12‐week results from the tolerance and effect of antipsychotics in children and adolescents with psychosis (TEA) trial. J Am Acad Child Adolesc Psychiatry. 2019;58(11):1062‐1078. 30858012 10.1016/j.jaac.2019.01.015

[39] Berling I , Isbister GK . Prolonged QT risk assessment in antipsychotic overdose using the QT nomogram. Ann Emerg Med. 2015;66(2):154‐164. 25639523 10.1016/j.annemergmed.2014.12.005

[40] Poluzzi E , Raschi E , Koci A , et al. Antipsychotics and torsadogenic risk: signals emerging from the US FDA adverse event reporting system database. Drug Saf. 2013;36(6):467‐479. 23553446 10.1007/s40264-013-0032-z PMC3664739

[41] Meyer‐Massetti C , Vaerini S , Rätz Bravo AE , Meier CR , Guglielmo BJ . Comparative safety of antipsychotics in the WHO pharmacovigilance database: the haloperidol case. Int J Clin Pharm. 2011;33(5):806‐814. 21809143 10.1007/s11096-011-9541-y

[42] Vieweg WV , Hasnain M , Howland RH , et al. Citalopram, QTc interval prolongation, and torsade de pointes. How should we apply the recent FDA ruling? Am J Med. 2012;125(9):859‐868. 22748401 10.1016/j.amjmed.2011.12.002

[43] Moore PW , Donovan JW , Burkhart KK , Haggerty D . A case series of patients with lamotrigine toxicity at one center from 2003 to 2012. Clin Toxicol (Phila). 2013;51(7):545‐549. 23869656 10.3109/15563650.2013.818685

