# Supplementary material for: Cardiac adverse events associated with quetiapine: Disproportionality analysis of FDA adverse event reporting system
Source: CNS Neurosci Ther. 2023 Apr 10;29(9):2705–16. doi: 10.1111/cns.14215 (PMC10401141; doi:10.1111/cns.14215)
Supplement: Supplementary file 1 — Data S1. [file CNS-29-2705-s001.docx]

**Supplementary Table S1**. Calculation of reporting odds ratio (ROR).

|  | Reports with target AE | Reports without target AE |
| --- | --- | --- |
| Reports with quetiapine | a | b |
| Reports without quetiapine | c | d |

a, number of reports containing both the target drug (quetiapine) and target AE; b, number of reports containing other AEs of the target drug; c, number of reports containing the target AE of other drugs; d, number of reports containing other drugs and other AEs.

AEs, Adverse Events; ROR, Reporting Odds Ratio; CI, confidence interval.

The calculation formulas are shown below:

1. ROR=ad/b/c
2. 95%CI=e^ln(ROR)±1.96(1/a+1/b+1/c+1/d)^0.5^

**Supplementary Table S2**. A rating scale assessing clinical priority of disproportionality signals.

| **Assessment items** | **2 points** | **1 point** | **0 point** |
| --- | --- | --- | --- |
| Number of target events | >50 | 10-50 | <10 |
| ROR_025_ | >5 | 2-5 | 1-2 |
| Mortality proportion | >50% | 25-50% | <25% |
| IMEs or DMEs | DME | IME | None |
| Relevant evidence evaluation | ++ | + | **-** |

Mortality proportion: percentage of cases in which death was reported as an outcome in the overall cases report for a particular adverse event. IMEs and DMEs are developed and updated by EMA (European Medicines Agency, 2020). ++ : AEs are mainly from the FDA Prescribing Information, the Summary of Product Characteristics of quetiapine posted by the MHRA, Phase 2/3 RCTs, or systematic reviews, with biological plausibility. + : AEs are mainly from other clinical trials, observational studies, or case reports/series with potential biological plausibility. - : AEs only emerging from disproportionality analyses.

AEs, Adverse Events; DMEs, Designated Medical Events; IMEs, Important Medical Events; MHRA, Medicine and Healthcare Products Regulatory Agency; RCTs, Randomized Controlled Trials; ROR_025_, the lower limit of 95% confidence interval of ROR.

**Supplementary Table S3.** Reporting odds ratios (ROR) with 95% CI for all non-[positive](javascript:;) quetiapine-related cardiac AEs.

| Adverse events | Quetiapine  (N=6,170), n | Non-Quetiapine  (N=6,431,012), n | ROR (95%Cl) |
| --- | --- | --- | --- |
| Cardiac disorders | 1,004 | 702,503 | 1.58 (1.48-1.70) |
| Dyspnoea | 116 | 168,608 | 0.71 (0.59-0.86) |
| Chest pain | 43 | 46,275 | 0.97 (0.72-1.31) |
| Oedema peripheral | 30 | 24,952 | 1.25 (0.88-1.80) |
| Peripheral swelling | 28 | 58,821 | 0.49 (0.34-0.72) |
| Myocardial infarction | 22 | 29,786 | 0.77 (0.51-1.17) |
| Chest discomfort | 22 | 29,311 | 0.78 (0.51-1.19) |
| Cardiac failure | 19 | 23,755 | 0.83 (0.53-1.31) |
| Pulmonary oedema | 14 | 12,458 | 1.17 (0.69-1.98) |
| Cardiac disorder | 14 | 25,546 | 0.57 (0.34-0.96) |
| Atrial fibrillation | 14 | 29,226 | 0.50 (0.29-0.84) |
| Angina pectoris | 12 | 7,434 | 1.68 (0.96-2.97) |
| Pericardial effusion | 8 | 6,458 | 1.29 (0.65-2.58) |
| Cardiac failure congestive | 8 | 13,074 | 0.64 (0.32-1.28) |
| Ascites | 8 | 8,232 | 1.01 (0.51-2.03) |
| Presyncope | 6 | 6,851 | 0.91 (0.41-2.03) |
| Sudden death | 5 | 2,336 | 2.23 (0.93-5.37) |
| Left ventricular dysfunction | 5 | 2,178 | 2.39 (1.00-5.76) |
| Cardiotoxicity | 5 | 3,103 | 1.68 (0.70-4.04) |
| Acute myocardial infarction | 5 | 6,560 | 0.79 (0.33-1.91) |
| Transposition of the great vessels | 4 | 160 | 26.07 (9.66-70.35) |
| Peripartum cardiomyopathy | 4 | 47 | 88.76 (31.97-246.44) |
| Pericarditis | 4 | 4,383 | 0.95 (0.36-2.54) |
| Hyperdynamic left ventricle | 4 | 73 | 57.15 (20.88-156.41) |
| Conduction disorder | 4 | 411 | 10.15 (3.79-27.18) |
| Bradycardia neonatal | 4 | 392 | 10.64 (3.97-28.51) |
| Atrioventricular block first degree | 4 | 1,161 | 3.59 (1.35-9.59) |
| Atrioventricular block complete | 4 | 1,688 | 2.47 (0.93-6.59) |
| Pulseless electrical activity | 3 | 1,477 | 2.12 (0.68-6.58) |
| Mitral valve incompetence | 3 | 2,453 | 1.27 (0.41-3.96) |
| Hypoplastic left heart syndrome | 3 | 139 | 22.51 (7.17-70.66) |
| Heart disease congenital | 3 | 949 | 3.30 (1.06-10.24) |
| Haemoptysis | 3 | 8,558 | 0.37 (0.12-1.13) |
| Coronary artery occlusion | 3 | 2718 | 1.15 (0.37-3.57) |
| Congestive hepatopathy | 3 | 352 | 8.89 (2.85-27.7) |
| Cardiomegaly | 3 | 2,569 | 1.22 (0.39-3.78) |
| Bundle branch block left | 3 | 1,101 | 2.84 (0.91-8.82) |
| Bradycardia foetal | 3 | 242 | 12.93 (4.14-40.37) |
| Atrioventricular block | 3 | 2,154 | 1.45 (0.47-4.51) |
| Ventricular hypertrophy | 2 | 242 | 8.62 (2.14-34.66) |
| Tachycardia induced cardiomyopathy | 2 | 54 | 38.62 (9.41-158.42) |
| Sinus bradycardia | 2 | 2,643 | 0.79 (0.20-3.16) |
| Pulmonary valve stenosis congenital | 2 | 104 | 20.05 (4.95-81.26) |
| Neonatal tachycardia | 2 | 29 | 71.91 (17.15-301.42) |
| Myocardial ischaemia | 2 | 2,117 | 0.98 (0.25-3.94) |
| Left ventricular hypertrophy | 2 | 1,101 | 1.89 (0.47-7.58) |
| Junctional ectopic tachycardia | 2 | 36 | 57.92 (13.94-240.63) |
| Hypervolaemia | 2 | 4,668 | 0.45 (0.11-1.79) |
| Endocarditis | 2 | 1,536 | 1.36 (0.34-5.43) |
| Atrial flutter | 2 | 2,302 | 0.91 (0.23-3.62) |
| Acute pulmonary oedema | 2 | 1,373 | 1.52 (0.38-6.08) |
| Wolff-Parkinson-White syndrome | 1 | 118 | 8.83 (1.23-63.25) |
| Ventricular dysfunction | 1 | 465 | 2.24 (0.32-15.95) |
| Supraventricular extrasystoles | 1 | 697 | 1.50 (0.21-10.63) |
| Sudden cardiac death | 1 | 560 | 1.86 (0.26-13.24) |
| Stress cardiomyopathy | 1 | 1,754 | 0.59 (0.08-4.22) |
| Sinus node dysfunction | 1 | 698 | 1.49 (0.21-10.62) |
| Sinus arrhythmia | 1 | 287 | 3.63 (0.51-25.88) |
| Right ventricular false tendon | 1 | 12 | 86.87 (11.29-668.23) |
| Pulmonary valve stenosis | 1 | 151 | 6.90 (0.97-49.34) |
| Pulmonary congestion | 1 | 3,657 | 0.28 (0.04-2.02) |
| Postural orthostatic tachycardia syndrome | 1 | 312 | 3.34( 0.47-23.80) |
| Pericardial haemorrhage | 1 | 709 | 1.47 (0.21-10.45) |
| Orthostatic intolerance | 1 | 292 | 3.57 (0.50-25.43) |
| Nodal rhythm | 1 | 309 | 3.37 (0.47-24.03) |
| Neonatal dyspnoea | 1 | 14 | 74.46 (9.79-566.36) |
| Mitral valve atresia | 1 | 26 | 40.09 (5.44-295.52) |
| Left ventricular enlargement | 1 | 148 | 7.04 (0.99-50.34) |
| Left atrial enlargement | 1 | 245 | 4.25 (0.60-30.33) |
| Hypersensitivity myocarditis | 1 | 47 | 22.18 (3.06-160.79) |
| Foetal heart rate disorder | 1 | 46 | 22.66 (3.12-164.36) |
| Fallot's tetralogy | 1 | 209 | 4.99 (0.70-35.58) |
| Eosinophilic myocarditis | 1 | 157 | 6.64 (0.93-47.44) |
| Ectopia cordis | 1 | 24 | 43.44 (5.88-321.14) |
| Dyspnoea exertional | 1 | 12,780 | 0.08 (0.01-0.58) |
| Dyspnoea at rest | 1 | 1,297 | 0.80 (0.11-5.71) |
| Dizziness exertional | 1 | 224 | 4.65 (0.65-33.19) |
| Dilatation atrial | 1 | 97 | 10.75 (1.50-77.08) |
| Cyanosis central | 1 | 100 | 10.42 (1.45-74.75) |
| Coronary artery stenosis | 1 | 902 | 1.16 (0.16-8.21) |
| Congenital pulmonary valve disorder | 1 | 23 | 45.32 (6.12-335.68) |
| Congenital pulmonary valve atresia | 1 | 38 | 27.43 (3.77-199.84) |
| Cardiovascular somatic symptom disorder | 1 | 12 | 86.87 (11.29-668.23) |
| Cardiovascular insufficiency | 1 | 478 | 2.18 (0.31-15.52) |
| Cardiopulmonary failure | 1 | 940 | 1.11 (0.16-7.88) |
| Cardiac valve disease | 1 | 1,537 | 0.68 (0.10-4.82) |
| Cardiac septal defect | 1 | 215 | 4.85 (0.68-34.58) |
| Cardiac dysfunction | 1 | 1,715 | 0.61 (0.09-4.32) |
| Cardiac discomfort | 1 | 711 | 1.47 (0.21-10.42) |
| Bundle branch block | 1 | 208 | 5.01 (0.70-35.75) |
| Bradyarrhythmia | 1 | 465 | 2.24 (0.32-15.95) |
| Bicuspid aortic valve | 1 | 99 | 10.53 (1.47-75.51) |
| Atrioventricular septal defect | 1 | 118 | 8.83 (1.23-63.25) |
| Atrioventricular block second degree | 1 | 749 | 1.39 (0.20-9.89) |
| Aortic valve sclerosis | 1 | 117 | 8.91 (1.24-63.80) |
| Aortic valve atresia | 1 | 27 | 38.61 (5.25-284.19) |
| Angina unstable | 1 | 1,182 | 0.88 (0.12-6.27) |
| Acute coronary syndrome | 1 | 2,045 | 0.51 (0.07-3.62) |

N/n, number of cases. ROR, reporting odds ratio; CI, confidence interval.

**Supplementary Table S4.** Signal strength of reports of quetiapine at the System Organ Class (SOC) level in FAERS database.

| System Organ Class (SOC) | Quetiapine cases  reporting SOC | ROR(95% CI) |
| --- | --- | --- |
| Psychiatric disorders* | 2,981 | 6.13 (5.83-6.44) |
| Nervous system disorders* | 2,910 | 3.50 (3.33-3.68) |
| Injury, poisoning and procedural complications* | 2,612 | 1.74 (1.66-1.83) |
| General disorders and administration site conditions* | 2,500 | 1.08 (1.03-1.14) |
| Investigations* | 1,087 | 1.65 (1.55-1.76) |
| Cardiac disorders* | 1,004 | 1.58 (1.48-1.70) |
| Vascular disorders | 892 | 0.97 (0.91-1.05) |
| Gastrointestinal disorders | 822 | 0.70 (0.65-0.75) |
| Musculoskeletal and connective tissue disorders* | 811 | 1.12 (1.04-1.20) |
| Metabolism and nutrition disorders* | 781 | 1.93 (1.79-2.08) |
| Respiratory, thoracic and mediastinal disorders | 772 | 0.85 (0.78-0.91) |
| Skin and subcutaneous tissue disorders | 457 | 0.41 (0.37-0.45) |
| Pregnancy, puerperium and perinatal conditions* | 397 | 3.65 (3.30-4.04) |
| Endocrine disorders* | 374 | 2.55 (2.30-2.83) |
| Renal and urinary disorders | 315 | 0.68 (0.61-0.76) |
| Infections and infestations | 290 | 0.38 (0.34-0.43) |
| Eye disorders | 277 | 1.06 (0.94-1.20) |
| Reproductive system and breast disorders | 276 | 0.95 (0.84-1.07) |
| Blood and lymphatic system disorders | 231 | 0.72 (0.63-0.82) |
| Immune system disorders | 226 | 0.35 (0.30-0.40) |
| Hepatobiliary disorders | 174 | 1.02 (0.88-1.18) |
| Social circumstances* | 122 | 1.59 (1.33-1.91) |
| Product issues | 114 | 0.42 (0.35-0.50) |
| Congenital, familial and genetic disorders* | 93 | 2.53 (2.06-3.10) |
| Ear and labyrinth disorders | 90 | 1.18 (0.96-1.45) |
| Neoplasms benign, malignant and unspecified | 84 | 0.13 (0.11-0.17) |
| Surgical and medical procedures | 53 | 0.25 (0.19-0.33) |

* indicates statistically significant signals in algorithm. ROR, reporting odds ratio; CI, confidence interval.

**Supplementary Table S5.** Reporting odds ratios (ROR) with 95% CI for sensitivity analysis.

| Adverse events | Quetiapine | Non-Quetiapine | ROR (95%Cl) |
| --- | --- | --- | --- |
| Cardiac disorders | 868 | 702,639 | 1.33 (1.24-1.43) |
| Dizziness | 173 | 135,011 | 1.35 (1.16-1.56) |
| Tachycardia | 120 | 25,705 | 4.94 (4.12-5.92) |
| Syncope | 55 | 28,368 | 2.03 (1.56-2.65) |
| Palpitations | 52 | 31,904 | 1.70 (1.30-2.24) |
| Cardio-respiratory arrest | 51 | 9,935 | 5.39 (4.09-7.10) |
| Cardiac arrest | 43 | 20,281 | 2.22 (1.64-3.00) |
| Sinus tachycardia | 41 | 3,673 | 11.71 (8.60-15.94) |
| Bradycardia | 33 | 16,683 | 2.07 (1.47-2.91) |
| Arrhythmia | 31 | 13,234 | 2.45 (1.72-3.49) |
| Myocarditis | 20 | 3,647 | 5.73 (3.69-8.90) |
| Atrial septal defect | 16 | 2,220 | 7.53 (4.60-12.32) |
| Cardiogenic shock | 13 | 4,070 | 3.33 (1.93-5.75) |
| Torsade de pointes | 13 | 2,086 | 6.51 (3.77-11.23) |
| Cyanosis | 10 | 3,648 | 2.86 (1.54-5.32) |
| Dizziness postural | 10 | 2,686 | 3.89 (2.09-7.23) |
| Cardiomyopathy | 9 | 3,690 | 2.54 (1.32-4.90) |
| Ventricular tachycardia | 9 | 3,912 | 2.40 (1.25-4.62) |
| Cardiovascular disorder | 8 | 3,694 | 2.26 (1.13-4.52) |
| Ventricular extrasystoles | 8 | 2,291 | 3.64 (1.82-7.30) |
| Ventricular fibrillation | 8 | 2,544 | 3.28 (1.64-6.57) |
| Tricuspid valve incompetence | 7 | 1,594 | 4.58 (2.18-9.63) |
| Brugada syndrome | 6 | 293 | 21.36 (9.52-47.96) |
| Congestive cardiomyopathy | 6 | 1,402 | 4.46 (2.00-9.96) |
| Patent ductus arteriosus | 6 | 983 | 6.37 (2.85-14.21) |
| Ventricular septal defect | 6 | 1,504 | 4.16 (1.87-9.28) |
| Tachyarrhythmia | 5 | 729 | 7.15 (2.97-17.25) |

ROR, reporting odds ratio; CI, confidence interval.
